# *Indonaiarectangularis* (Tapparone-Canefri, 1889), comb. nov., a forgotten freshwater mussel species from Myanmar (Bivalvia, Unionidae)

**DOI:** 10.3897/zookeys.852.33898

**Published:** 2019-06-05

**Authors:** Ivan N. Bolotov, Ilya V. Vikhrev, Manuel Lopes-Lima, Mikhail Y. Gofarov, Ekaterina S. Konopleva, Zau Lunn, Nyein Chan, Arthur E. Bogan

**Affiliations:** 1 Northern Arctic Federal University, Northern Dvina Emb. 17, 163002 Arkhangelsk, Russian Federation Northern Arctic Federal University Arkhangelsk Russia; 2 Federal Center for Integrated Arctic Research, Russian Academy of Sciences, Northern Dvina Emb. 23, 163000 Arkhangelsk, Russian Federation Federal Center for Integrated Arctic Research, Russian Academy of Sciences Arkhangelsk Russia; 3 CIBIO/InBIO – Research Center in Biodiversity and Genetic Resources, University of Porto, Campus Agrário de Vairão, Rua Padre Armando Quintas 7, 4485-661 Vairão, Portugal University of Porto Porto Portugal; 4 CIIMAR/CIMAR – Interdisciplinary Centre of Marine and Environmental Research, University of Porto, Terminal de Cruzeiros do Porto de Leixões, Avenida General Norton de Matos, S/N, 4450-208 Matosinhos, Portugal International Union for Conservation of Nature Cambridge United Kingdom; 5 SSC/IUCN – Mollusc Specialist Group, Species Survival Commission, International Union for Conservation of Nature, c/o The David Attenborough Building, Pembroke Street, CB2 3QZ Cambridge, UK Fauna & Flora International – Myanmar Program Yangon Myanmar; 6 Fauna & Flora International – Myanmar Program, Yangon, Myanmar Fauna & Flora International – Myanmar Program Yangon Myanmar; 7 North Carolina State Museum of Natural Sciences, 11 West Jones St., Raleigh, NC 27601, USA North Carolina State Museum of Natural Sciences Raleigh United States of America

**Keywords:** Ayeyarwady River, Indochinellini, Margaritiferidae, Parreysiinae, Southeast Asia, Unionidae

## Abstract

*Uniorectangularis* Tapparone-Canefri, 1889 is a little-known nominal species of freshwater mussels described from a tributary of the Ayeyarwady River in Myanmar. This taxon was considered a synonym of *Gibbosulalaosensis* (Lea, 1863), a margaritiferid species. However, the range of *Gibbosulalaosensis* does not encompass the Ayeyarwady River watershed. Here we re-examine the holotype of *Uniorectangularis* and provide a conchological re-description of this species. Based on conchological features such as the shell shape, elevated umbo, and the structure of lateral and pseudocardinal teeth, we transfer this taxon to the genus *Indonaia* Prashad, 1918 and propose *I.rectangularis* (Tapparone-Canefri, 1889), **comb. nov.** It appears to be a rare freshwater mussel species with a restricted range, because it has not been found since the original description. Two additional species in this genus are known from Myanmar, i.e. *Indonaiaandersoniana* (Nevill, 1877) and *I.subclathrata* (Martens, 1899).

## Introduction

*Gibbosulalaosensis* (Lea, 1863) (Bivalvia, Margaritiferidae) is an endangered freshwater mussel species from Southeast Asia (Bolotov et al. 2014), which has recently been transferred from *Margaritifera* Schumacher, 1816 to *Gibbosula* Simpson, 1900 (Lopes-Lima et al. 2018). Several enigmatic nominal taxa and nomina dubia were linked to this species as its putative synonyms or closely related species, i.e. *Uniorectangularis* Tapparone-Canefri, 1889, *U.sula* Simpson, 1900, *U.sella* Haas, 1912, and *Margaritanopsiswoodthorpi* Godwin-Austen, 1919 (Simpson 1900; Haas 1912; Godwin-Austen 1919; Prashad 1922). While the complete story of the three latter names remains to be discussed, a taxonomic reassignment of *Uniorectangularis* is presented here.

Leonardo Fea, an adventurous Italian pioneer and explorer, found a single shell of *Uniorectangularis* among large collections of non-marine molluscs from British Burma during his travels in 1885–1887 (Tapparone-Canefri 1889; Prashad 1922). Cesare Maria Tapparone-Canefri, a famous Italian malacologist, studied this sample and published a comprehensive paper (Tapparone-Canefri 1889) with a description of numerous new taxa of terrestrial and freshwater molluscs, including *Uniorectangularis*.

Later, Prashad (1922) revisited Tapparone-Canefri’s unionid taxa in his broad-scale review of freshwater mussels described from British Burma. In the account on *Gibbosulalaosensis*, Prashad (1922: 93) stated that: “The species described as *Uniorectangularis* by Tapparone-Canefri [...] is based on a single very young shell. It is undoubtedly to be referred to the genus *Margaritanopsis* and probably represents another species of the genus. Owing, however, to a single young shell being available I do not feel disposed to consider it as a distinct species…”. After that generic reassignment of *Uniorectangularis*, this species was completely forgotten, lost to malacological taxonomy, and it was not listed in the subsequent taxonomic checklists on freshwater mussels of the Oriental Region (Brandt 1974; Subba-Rao 1989; Bolotov et al. 2017a; Zieritz et al. 2018) and in the most authoritative global revisions (Haas 1969; Graf and Cummings 2007).

We revise the generic placement of *Uniorectangularis* and discuss its prospective taxonomic status based on morphological study of the type specimen.

## Material and methods

We studied the holotype of *Uniorectangularis* in the Museo Civico di Storia Naturale di Genovay (MSNG), Genova, Italy. The images of the specimen were taken with Canon EOS 7D DSLR camera (Canon Inc., Japan). Shell measurements were performed with Adobe Photoshop CS using digital photographs of the holotype. Samples of *Indonaiaandersoniana* (Nevill, 1877) (*N* = 37 specimens) and *I.subclathrata* (Martens, 1899) (*N* = 7 specimens) were studied in the Russian Museum of Biodiversity Hotspots (RMBH), Federal Center for Integrated Arctic Research of the Russian Academy of Sciences, Arkhangelsk, Russia. Three shell dimensions at each specimen of the studied taxa (length, height, and width of the shell, all taken at the maximum diameter) were measured using calipers (±0.1 mm). The comparative analysis of shell morphology was carried out with regard to the main distinguishing traits, such as shell shape, umbo position, structures of pseudocardinal and lateral teeth, as well as muscle attachment scars (Konopleva et al. 2019).

## Results

### Family Unionidae Rafinesque, 1820

#### Subfamily Parreysiinae Henderson, 1935

##### Tribe Indochinellini Bolotov, Pfeiffer, Vikhrev & Konopleva, 2018

###### Genus *Indonaia* Prashad, 1918

####### Indonaia rectangularis

Taxon classificationAnimaliaUnionidaUnionidae

(Tapparone-Canefri, 1889) comb. nov.

Figure 1A–F

=UniorectangularisTapparone-Canefri 1889: 354.
=Margaritanopsisrectangularis (Tapparone-Canefri, 1889). – Prashad 1922: 93, pl. 2, fig. 5.

######## Type.

**Figure 1.**
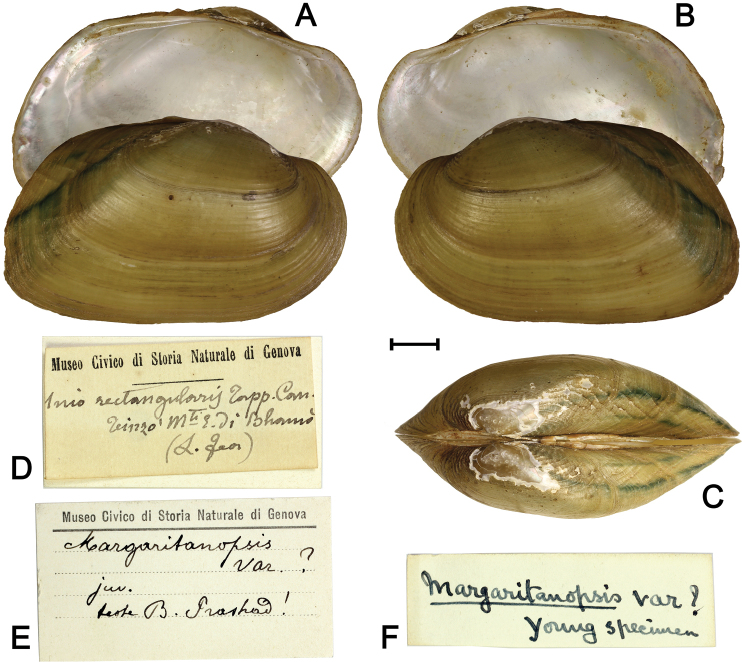
Holotype of *Indonaiarectangularis* (Tapparone-Canefri, 1889), comb. nov. [MSNG]. **A, B** Shell, lateral view: inner side of the left valve and outer side of the right valve (**A**); vice versa (**B**) **C** shell, dorsal view **D** original label [probably by C.M. Tapparone-Canefri] **E, F** secondary labels [probably by B. Prashad]. Scale bar: 5 mm. (Photos: Ilya V. Vikhrev).

Holotype in MSNG, without ID number (Fig. 1A–C). Original label: “*Uniorectangularis* Tapp. Can. Teinzo, Mti E. di Bhamo (L. Fea)” (Fig. 1D). Secondary labels: “*Margaritanopsis* var.? juv. detto B. Prashad!” (Fig. 1E) and “*Margaritanopsis* var.? Young specimen” (Fig. 1F). The original label does not have a collecting date, but Fea’s sample of another freshwater mussel species from Teinzo residing in the MSNG is dated “Marzo 1886” that may also be applicable to the holotype of *U.rectangularis*.

######## Type locality.

Teinzo (presently Teinthaw village), 24.3978N, 97.2519E, Moolay River (Mole Chaung in Burmese), hills E of Bhamo (L. Fea), alt. 110 m a.s.l., Ayeyarwady Basin, Myanmar (Tapparone-Canefri 1889).

######## Diagnosis.

**Figure 2.**
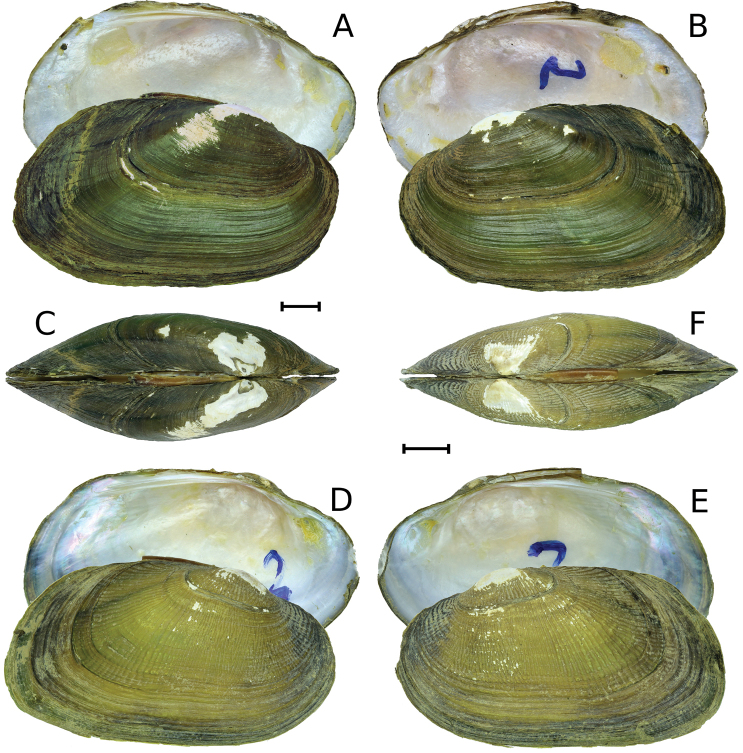
Specimens of *Indonaiaandersoniana* (Nevill, 1877) and *I.subclathrata* (Martens, 1899) from Myanmar [RMBH biv450_2 and RMBH biv347_2, respectively]. **A, B** Shell of *I.andersoniana*, lateral view: inner side of the left valve and outer side of the right valve (**A**); vice versa (**B**) **C** shell of *I.andersoniana*, dorsal view **D, E** shell of *I.subclathrata*, lateral view: inner side of the left valve and outer side of the right valve (**D**); vice versa (**E**) **F** shell of *I.subclathrata*, dorsal view. Scale bar: 5 mm. (Photos: Ekaterina S. Konopleva).

As Tapparone-Canefri (1889) stated, the shell of *Indonaiarectangularis* is not similar to any other freshwater mussel species known from Myanmar. This species cannot be mistaken with the two congeners, *Indonaiaandersoniana* and *I.subclathrata* (Fig. 2), that were described from the Ayeyarwady Basin. Both these species differ from *Indonaiarectangularis* by a more elongated shell and less developed lateral, and pseudocardinal teeth. The pseudocardinal teeth in *I.rectangularis* are lamellar, thick, and very elongated. Additionally, a unique feature of *I.rectangularis* is the presence of regular ridges crossing the growth lines in the posterior-dorsal area forming a clear rectangular pattern that was never seen in other species (Fig. 1C).

######## Redescription.

Shell length 34.2 mm, height 20.4 mm, width 16.2 mm. Shell thick, obovate, inequilateral, with broader posterior side. Dorsal margin slightly convex. Ventral margin nearly straight. Anterior margin rounded. Posterior margin slightly elevated posteriorly, with an inconspicuous wing. Umbo prominent, elevated, rounded, slightly eroded. Shell surface mostly smooth. In the posterior-dorsal area, regular ridges cross the growth lines and form a clear rectangular pattern. In the anterior-dorsal area, curved, lamella-like ridges closely spaced along growth lines. Periostracum light olive-brown, with two parallel, slightly curved green bands along posterior-dorsal area; inner band with a broad greenish extension posteriorly. Nacre silver-white. Umbo cavity deep. Anterior adductor scar round, shallow but well marked. Posterior adductor scar oval, very shallow, unclear. Mantle attachment scars absent. Pseudocardinal teeth are thick, lamella-like, very elongated, two teeth in the right valve and one tooth in the left valve. Lateral teeth well developed, thick, elongated and straight, one tooth in the right valve and two teeth in the left valve. Soft body morphology and anatomy unknown.

######## Remarks.

*Uniorectangularis* was originally described based on a single specimen with a shell 34.2 mm long, 20 mm high and 16 mm wide (Tapparone-Canefri 1889). The single shell labelled as *Uniorectangularis* and deposited in the MSNG studied here corresponds in dimensions to the specimen described by Tapparone-Canefri. The original label (Fig. 1D) also affirms that this shell is the holotype of *Uniorectangularis* designated by monotypy.

######## Distribution.

**Figure 3.**
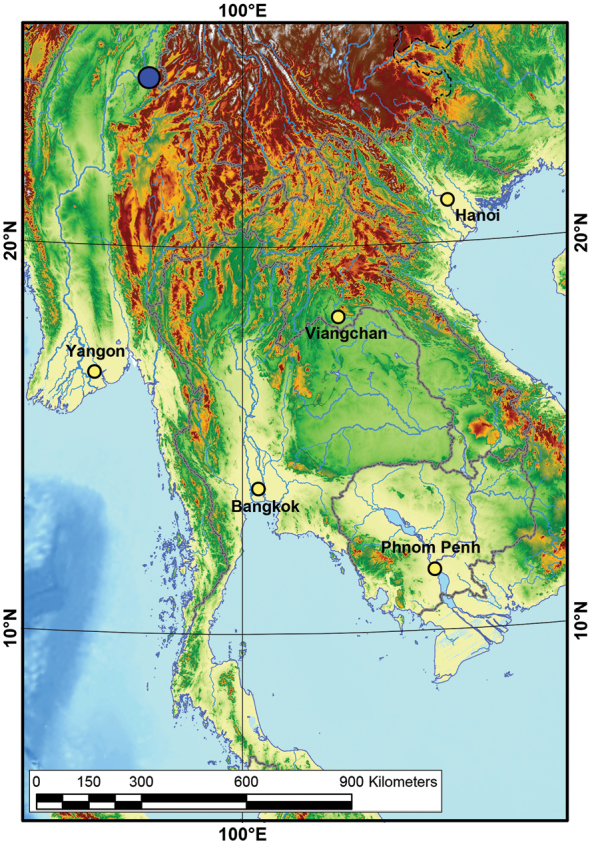
Map of the type locality of *Indonaiarectangularis* (Tapparone-Canefri, 1889), comb. nov. (dark blue circle). The digital elevation model and other layers of the map were added from the Esri Data & Maps 10 dataset.

This species is known only from the type locality, a tributary of the Ayeyarwady River (Fig. 3). It appears to be a rather restricted and rare species, because it has not been recorded since the original description, even during a recent broad-scale survey of freshwater mussels in Myanmar (Bolotov et al. 2017a, b, 2018; Konopleva et al. 2019). However, there has been no studies on freshwater mussels from the Mole River published since Tapparone-Canefri (1889).

## Discussion

The results of our study reveal that *Uniorectangularis* is not a margaritiferid because it does not have mantle attachment scars, the most prominent diagnostic feature of the Margaritiferidae (Lopes-Lima et al. 2018). *Uniorectangularis* can be placed within the genus *Indonaia* based on conchological features, i.e. the shell shape, elevated umbo, and the structure of lateral and pseudocardinal teeth (Figs 1, 2). Prashad (1922) considered that the holotype of *Uniorectangularis* is a “very young shell”. However, all *Indonaia* species are rather small mussels (Fig. 2), and this holotype shell surely represents an adult specimen.

*Indonaia* represents the most divergent phylogenetic clade among the tribe Indochinellini (Bolotov et al. 2017a, 2018; Pfeiffer et al. 2018; Konopleva et al. 2019). This genus contains three species from India and three species from Myanmar (Konopleva et al. 2019). The taxonomic relationship of *Indonaia* with another Oriental genus, *Radiatula* Simpson, 1900, is still to be resolved, because molecular sequences of *Uniocrispisulcatus* Benson, 1862, the type species of *Radiatula*, are still not available.

### Key to species of *Indonaia* from Myanmar

**Table:** 

1	Shell surface with radial ridges covering the entire shell disc	*I.subclathrata* (Martens, 1899)^*^
–	Shell surface without radial ridges on the shell disc	**2**
2	Shell surface mostly smooth, while regular ridges cross the growth lines and form a clear rectangular pattern in the posterior-dorsal area, and curved, lamella-like ridges closely spaced along growth lines in the anterior-dorsal area	*I.rectangularis* (Tapparone-Canefri, 1889), comb. nov.^**^
–	Shell surface smooth	*I.andersoniana* (Nevill, 1877)^***^

## Supplementary Material

XML Treatment for Indonaia rectangularis

## Footnotes

Manipur, Chindwin rivers, middle reaches of the Ayeyarwady River; common species (Konopleva et al. 2019)

Mole River, Ayeyarwady Basin; rare species

Middle reaches and downstream of the Ayeyarwady River; common species (Bolotov et al. 2017a, 2018; Konopleva et al. 2019)
